# Public perceptions of health data sharing for artificial intelligence research: a qualitative focus group study in the UK

**DOI:** 10.1136/bmjdhai-2025-000239

**Published:** 2026-02-09

**Authors:** Rachel Kuo, Ciarán O’Hanlon, Judi Smith, Rosie Hill, Bartlomiej W Papiez, Gary S Collins, Dominic Furniss, Elizabeth Tutton

**Affiliations:** 1 Nuffield Department of Orthopaedics, Rheumatology, and Musculoskeletal Sciences, University of Oxford, Oxford, UK; 2 Department of Plastic and Reconstructive Surgery, John Radcliffe Hospital, Oxford, UK; 3 University of Oxford Big Data Institute, Oxford, UK; 4 Department of Applied Health Sciences, School of Health Sciences, University of Birmingham, Birmingham, UK; 5 NIHR Birmingham Biomedical Research Centre, University Hospitals Birmingham NHS Foundation Trust and University of Birmingham, NIHR, London, UK

**Keywords:** Artificial intelligence, Public Health, Health Services Research, Patient Involvement, Health Communication

## Abstract

**Objective:**

Artificial intelligence (AI) in healthcare offers potential to improve diagnosis, patient care and system efficiency. However, AI development, evaluation and post-implementation monitoring require large volumes of health data, and public trust is essential for enabling such data sharing. This study aimed to explore UK public perceptions of health data sharing for AI research, and to identify factors influencing willingness to participate.

**Methods and analysis:**

We conducted eight 90-min online focus groups in May–July 2024, with 41 purposively sampled adult (18 years or older) members of the UK public, recruited via a national registry and departmental social media. Groups were selected to maximise diversity in age, ethnicity, household income, education, health status and geography. We used thematic analysis to iteratively develop themes and subthemes inductively.

**Results:**

Three key themes were developed: (1) perceived general risks of health data sharing, including concerns about the limits of anonymisation, the sensitivity and scope of data requested, data governance and security, and trust in different data custodians. Participants viewed anonymisation as essential but fallible, especially for rare conditions or large linked datasets, and held a pragmatic view on sharing data with commercial organisations; (2) individual risk-benefit assessment, reflecting how people weighed potential personal harms, such as discrimination, data misuse and risks for children, against anticipated benefits, including altruism, improved care and the perceived clinical value of AI; (3) informed consent as a foundation for trust, encompassing preferences for clear, study-specific, tailored information about data use and AI purposes, and for consent processes that provided choice, avoided emotional pressure and allowed time for reflection or later withdrawal.

**Conclusion:**

Trust in health-data sharing for AI is conditional and shaped by participant-level and study-specific risks and benefits. These findings highlight public expectation for transparent governance, clear justification for data use and public benefit, particularly where commercial involvement is proposed. In the context of expanding healthcare digital infrastructure and emerging UK/European Union regulatory frameworks for AI governance and reporting, understanding these expectations will be essential to building and sustaining the social licence required for large-scale data use, supporting equitable participation and representation in AI research.

**WHAT IS ALREADY KNOWN ON THIS TOPIC**

- Prior qualitative studies have identified widespread concerns surrounding anonymisation, commercial exploitation, data security, artificial intelligence (AI) bias and the under-representation of minority groups in health datasets. These studies provide critical insight into public views on health data sharing for AI research. Our study builds on the literature by offering a detailed analysis of how members of the public weigh these concerns when deciding whether to share their health data, with practical implications for researchers and policymakers.

**WHAT THIS STUDY ADDS**

- This study provides in-depth insights from eight focus groups, with representation of ethnic minority groups in line with UK census data. We identify three themes that shape trust and willingness to participate: (1) perceived general risks of health data sharing; (2) individual risk-benefit assessment; and (3) informed consent as a foundation for trust. Participants emphasised concerns around the limits of anonymisation, tiered sensitivity of health data and the role of commercial organisations, while preferring personalised, transparent consent processes.

**HOW THIS STUDY MIGHT AFFECT RESEARCH, PRACTICE OR POLICY**

- Our findings underscore the necessity for robust and transparent governance frameworks to support health data sharing for AI research. Effective anonymisation, especially in the context of large-scale databases and database linkage, must be accompanied by clear accountability mechanisms and consequences for data breaches. Communication strategies should be tailored to public concerns, clearly stating the added value of AI in individual studies, the reason for the data requested and which entities will be using and profiting from the data. Consent processes should respect individual preferences, offering opportunities for personalisation, ‘cooling-off’ periods or study withdrawal. We recommend further international comparative studies to better understand global attitudes and inform culturally sensitive policies and practices and therefore promote equitable, representative participation in healthcare AI research.

## Introduction

Artificial intelligence (AI) within healthcare holds significant promise for improving diagnosis, patient management and system efficiency.^1 2^ AI tools are being developed across multiple clinical domains. Imaging-based AI systems have shown high diagnostic accuracy in silico for cancers, fractures and retinal disease, while natural language processing tools have been proposed to support information extraction from electronic health records (EHR) and automate medical documentation.^3–9^ Despite an expanding evidence base, adoption remains uneven. Within the UK, National Health Service (NHS) deployment is largely limited to locally implemented systems, while many AI systems remain at pilot or evaluation stage.^10 11^ Persistent challenges include variable data quality, limited data diversity and restricted availability of large-scale, labelled datasets.^12 13^

Stakeholder studies reveal cautious optimism about healthcare AI, tempered by concerns about public-private sector relationships, algorithmic bias and privacy.^13–15^ Because AI development, validation and monitoring require large, representative datasets, public confidence is central to the success of healthcare AI research. However, willingness to share health data is constrained by fears of commercial exploitation, re-identification and misuse of personal information.^16–19^ Specifically, willingness to share health data with commercial organisations has declined, influenced by high-profile data breaches, misuse and cyberattacks.^16 19–21^ Attitudes are shaped by intersecting personal, institutional and societal factors.^22–25^ Individuals from minority ethnic and low-income groups are less likely to engage in research, adopt EHRs and/or trust healthcare systems.^26–28^ Differential recruitment to studies between demographic groups risks limiting generalisability and exacerbating existing health inequalities.^29^

These concerns are recognised in recent international guidance, including the FUTURE-AI framework for trustworthy AI in healthcare, which emphasises dataset representativeness, stakeholder engagement and bias mitigation as core principles.^30^ The framework outlines six domains: fairness, universality, traceability, usability, robustness and explainability, intended to guide development and post-market monitoring.^30^ Translating these principles into practice requires public confidence in how health data are governed and used. Understanding public expectations is therefore essential to ensuring responsible and socially acceptable AI development.

This study explores public perceptions of health data sharing for AI healthcare research in the UK, identifying key determinants of trust and willingness to participate. We provide insights to inform strategies to enhance public acceptability and recruitment for AI-driven healthcare research.

## Methods

### Study design

We conducted a qualitative focus group study with adult members of the UK public to explore perceptions of health-data sharing for AI research. Focus groups were chosen to elicit interaction and debate among participants, who were purposively sampled for demographic diversity. The study was developed following a qualitative evidence synthesis (QES) exploring stakeholder perspectives towards diagnostic AI previously conducted by RK, JS, RH, GSC, DF and ET.^13^ Eight online focus groups were held between May and July 2024.

### Recruitment

Eligible participants were 18 years or older, fluent in English and had accessed UK NHS care at least once since 2005, when EHRs were first implemented. We recruited participants through the National Institute for Health Research ‘Be Part of Research Volunteer Service’ (NIHR-BPORVS) registry and department social media in April 2024. NIHR-BPORVS is a UK-wide healthcare registry, maintaining a list of 4929 members who have agreed to be contacted about research opportunities. Eligible registry members received an e-mail invitation from the NIHR-BPORVS. Social media posts directed potential participants to the study website, which provided the Participant Information Sheet and contact details for the study.^31^

Informed consent was obtained electronically. After consenting to take part, potential participants completed a demographic questionnaire. We purposively sampled for maximum variation in age, sex, ethnicity, education, household income, presence of a chronic health condition and geographical location within the UK, to capture a wide demographic. We invited participants to one of eight planned focus groups, maximising diversity in each group.

### Topic guide

The topic guide was developed based on previous qualitative and mixed-method evidence syntheses on stakeholder perceptions of AI.^13 15 19 32 33^ These studies highlighted variation in how AI was perceived and understood and widespread concerns about data security, regulation and commercial access. The guide included three scenarios to facilitate discussion (online supplemental appendix). Scenario 1 was designed to establish a baseline for discussion, describing consent to share limited, specific health data for a university-led research project focusing on cancer. Scenario 2 introduced broader, non-specific data-sharing with a research database, in a primary care mental health consultation. Scenario 3 focused on parental consent for a child, international data sharing and commercial involvement. The topic guide was piloted in mock focus groups (RK, CO, JS, RH) to assess clarity, relevance and timing.

10.1136/bmjdhai-2025-000239.supp1 Supplementary data

### Focus groups

Eight 90-min focus groups using Microsoft Teams video-conferencing software were conducted over a 6-week period in May–July 2024. Discussions were held online to increase geographical reach and flexibility for those with work, caring or health commitments. Discussions began with open-ended questions, followed by scenario-based discussions.

Sessions were video-recorded and transcribed verbatim via a secure third party.^34^ After transcription, video recordings were destroyed. Participants were offered £25 reimbursement.

### Data analysis

We conducted thematic analysis, using an inductive, data-driven approach.^35^ Analysis was iterative, aiming to identify patterns of shared meaning across the dataset.

Researchers took contemporaneous field notes and debriefed after each focus group. Next,^34^ anonymised transcripts were analysed by pairs of researchers (RK and one of CO/RH/JS), who independently familiarised themselves with the data. Codes were generated inductively and compared after every two focus groups. Discrepancies were resolved through discussion and consensus. The team reflected on how their experience and existing literature might shape interpretation.^13^ After the fourth focus group, a subgroup (RK, CO, RH, JS, ET) met to discuss code convergence and construct candidate themes. This collaborative approach continued until all focus groups were analysed. All co-authors were included in the final reflective discussion.

NVivo and Microsoft Word were used for analysis.^36^ Initially, Microsoft Word was used to annotate the transcripts and identify provisional codes. Following discussions between team members, NVivo was used to manage detailed coding of all transcripts and development of themes. The study was reported using the SRQR checklist (online supplemental appendix).^37^

### Researcher characteristics

Our research team comprised three clinician-researchers (RK, CO, DF), two patient and public involvement (PPI) and engagement partners (JS, RH), one senior qualitative researcher (ET), one computer scientist (BP) and a statistician/methodologist (GSC).

There were no pre-existing relationships between facilitators and participants. At the start of each group, facilitators introduced themselves and their roles. All authors provide reflexivity statements in the online supplemental appendix.

### PPI

Prior to this study, RK, JS, RH, GSC, DF, ET and a wider group of PPI co-producers published a QES of stakeholder perceptions of diagnostic AI, which identified the need for further research focussing on data sharing.^13^ RK, JS and RH conceived of this study in response to this gap. They co-designed the study, developed the topic guide, organised, recruited and facilitated focus groups; kept field notes; coded transcripts; developed themes and subthemes; and drafted the manuscript. JS has helped to present this work to a wider PPI group in Oxford University and will contribute to ongoing dissemination.

## Results

### Participants

Of 114 responses, 67 participants were invited, and 41 attended one of eight focus groups. We did not ask non-attenders to give a reason for their absence.

Twenty-two participants were female (online supplemental table 1). The mean age of participants was 56.7 years (range 23–78). Thirty-two participants were white, four were Indian/Asian, three were ‘other ethnic minority’, one was Black African and one was mixed-race. Thirty participants had a chronic health condition (over 3 months’ symptom duration). The median combined household income of participants was £50–69 000/year, ranging from <£10 000–>£130 000 /year. Twenty-five participants held an undergraduate degree or higher.

10.1136/bmjdhai-2025-000239.supp2 Supplementary data

### Scenario-based responses

Discussions in each focus group were structured around three scenarios. Across groups, participants were most comfortable sharing data in the first scenario, where the purpose was clearly defined, the data scope limited and the study university-led. The second prompted more ambivalence: while many recognised the potential value of large research databases, they expressed concern about loss of control and vulnerability to data misuse. The third scenario elicited the most caution, particularly due to consenting on behalf of children, international data transfers and commercial involvement. These differences in willingness to share data across scenarios informed the three themes described below.

### Themes

Three themes were developed to describe participants’ perceptions of health data sharing for AI research: (1) perceived general risks of health data sharing; (2) individual risk-benefit assessment; and (3) informed consent as a foundation for trust. The first and second themes reflect general and individual-level considerations influencing participants’ decision to share health data. The third describes methods by which researchers can support informed decision-making.

#### Perceived general risks of health data sharing

Participants highlighted systemic concerns about how health data might be handled, accessed and secured in the context of AI research. Four subthemes described these views: (A) the limits of anonymisation, in which participants discussed the concurrent importance and limitations of health record de-identification; (B) sensitivity and scope of shared data, where participants reflected on purposeful data use and personal control; (C) trust in data governance and security, centred on concerns surrounding data breaches, misuse and unclear accountability; and (D) perceptions of data custodianship and use, highlighting differing levels of trust across institutions and sectors.

##### The limits of anonymisation

Anonymisation was seen as essential to health data sharing, necessary to ‘protect the person behind the data’ (P4). Effective anonymisation meant that ‘someone that knew you wouldn’t be able to recognise you from what they were seeing’ (P2). Redacting explicit identifiers like names and addresses was viewed as reducing risk and making sharing ‘less intrusive’ (P15). For some, this alone made sharing acceptable: ‘It’s anonymous! They don’t know it’s me, I don’t care’ (P3).

Participants acknowledged anonymisation as a spectrum with potential for re-identification in rare medical conditions or through data linkage: ‘how anonymous, anonymous data is, is really hard to say, especially when it comes to healthcare and the more unusual the thing you’ve got is*’* (P39).

Concerns were raised about quality control, with calls for transparency around redacted methods and assurance processes: ‘how are we going to know it’s been successfully redacted?’ (P34). Others worried about future advances making re-identification feasible: ‘It may be anonymised now, but who’s to say in 10 years’ time, suddenly we’ll come up with a technology that enables you to decrypt the data?’ (P18). A minority saw potential benefits in re-identification, such as learning about study results, or uncovering personal health information about themselves during the research process.

##### Sensitivity and scope of shared data

Health data was viewed as particularly sensitive, carrying a ‘potentially huge emotional cost*’* (P40). Some diagnoses were viewed as particularly sensitive. Certain conditions, such as those related to mental health, sexually transmitted infections, reproductive health and obesity, were seen to ‘rightly or wrongly, have a stigma attached to them’ (P25), making affected individuals more vulnerable to misuse or exploitation.

Participants drew a clear distinction between sharing data for targeted research and broader, non-specific data. They saw more utility with specific, purposeful use: ‘There is no point to pouring it all in and then see what happens’ (P11). Sharing extensive personal information was associated with a strong feeling of loss of control: ‘every piece of my medical history…just out there somewhere. I would feel quite vulnerable and angry’ (P3). While participants were in principle willing to share large volumes of data, this required greater justification: ‘you would ask yourself why…how is it going to be used, and is it actually necessary?’ (P37).

There was tension between the preference for specificity and the belief that large, diverse datasets improve AI performance. Some felt the relevance of certain data might only emerge retrospectively: ‘We aren’t powerful enough yet to be able to analyse it all, but…we may have to accept in the future we plug ourselves into a machine and it takes all sorts of information off us. And I don’t know, maybe we’ll be healthier for it’ (P32).

##### Trust in data governance and security

All focus groups emphasised the importance of robust data management, ‘a structure that says this is how and why we do things and when things go wrong this is what we do’ (P36). Limiting access to authorised users was seen as essential to prevent errors or misuse, both accidental and deliberate: ‘Some people will, with good intentions, set out at the beginning and then they find, I can sell this on…I’m going to make some money out of it, and it all becomes too tempting’ (P27).

Citing personal experiences and media coverage, participants agreed that all data systems are vulnerable to hacking: ‘if you put data in, it will be hacked’ (P30). To prevent misuse or theft, participants advocated for ‘*checks and balances’* (P41), including regular audit and transparent protocols for reporting breaches. They emphasised accountability, including clear consequences for what were perceived to be inevitable data breaches: ‘How will complaints, concerns, whistleblowing or major issues be dealt with after the event?’ (P31).

Participants also stressed the need for clear policies on data retention and destruction. Uncertainty about whether derived data might persist indefinitely raised concerns: ‘It never says the data will not be copied…the data that will be generated from that data may continue to exist’ (P7). Explicit information on terms for data retention and study periods was crucial for acceptability. Indefinite or extended study periods were seen as increasing the risk of data misuse or breach.

##### Perceptions of data custodianship and use

Participants’ trust was closely tied to who would access the data, and where it would be stored. While healthcare providers were generally trusted, data leaving clinical systems raised concerns. Universities were viewed positively, perceived as serving the public interest. Profit-making by academic institutions was acceptable when aligned with broader societal benefit: ‘[University X] is notorious for making money with their start-ups. I see no problems with it because they can, with that money, continue to offer high quality education*’* (P3).

In contrast, general research databases prompted unease due to a perceived loss of control. Participants preferred targeted studies with strictly defined purposes: ‘Could anyone come along, come up with any idea and use the data…if there’s criteria for how the data’s going to be used, I’d be more comfortable’ (P36). Limiting access to named users and granting access to only the minimum necessary data were perceived to improve acceptability.

Commercial entities elicited the most scepticism, viewed as primarily profit-driven: ‘illness is money for them’ (P2). Participants worried about exploitation, data misuse and unequal contracts: ‘they make mincemeat of you if you’re not careful’ (P35). Even when commercial research aimed to advance healthcare AI, participants were suspicious of secondary motives: ‘[Company X] says, “OK, let’s make this AI for public good”, but the real reason is to gather as much data as possible…let’s sell that on and use it in our other businesses’ (P37).

Despite this, many described the value of commercial involvement in driving healthcare innovation, due to greater resources: ‘they will get it done…there’s big money involved here’ (P39). Participants generally accepted commercial involvement as transactional, contingent on patient benefit: ‘I don’t have concerns about companies making money as long as patients benefit’ (P35). This highlighted an important tension: while commercial organisations were generally regarded with greater suspicion than public-sector actors, views were pragmatic rather than uniformly negative. Participants described a highly conditional form of acceptance, in which commercial activity was seen as permissible only when governed by strict safeguards and demonstrably aligned with patient benefit. Here, commercial involvement was acceptable not because profit was welcomed, but because participants believed that commercial resources could accelerate AI research and serve the wider public interest: ‘If the company is happy, and in the end the patient and people benefit, the whole system benefits…so when it’s blended this way it’s all kind of a mutual benefit (P4)’.

International data sharing raised concerns about jurisdictional oversight and data security, but participants also recognised potential advantages, such as improved diversity, collaboration and funding opportunities: ‘there’s excellent research done elsewhere, and they might want global data to get the numbers up’ (P2). Participants called for information about involved countries, their data security practices, the rationale and potential benefit in sharing data.

#### Individual risk-benefit assessment

Participants made personal calculations about whether to share data, weighing perceived risks against potential benefits to themselves and others. Two subthemes were developed to capture this process: (A) personal considerations and perceptions of harms, describing participant worries about discrimination, criminal activity and implications for children and (B) anticipated benefits and motivations, highlighting altruism, personal experiences of healthcare and the perceived clinical value of AI technology.

##### Personal considerations and perception of harm

The individual level of risk was informed by personal characteristics and perception of technology. Participants were concerned about the misuse of health data by malicious individuals, leading to blackmail, scamming and identity fraud. They worried about institutional misuse and discrimination by employers and insurers. This was of particular concern for those of working age, and with pre-existing conditions related to sensitive health issues: ‘I have rapid cycle bipolar. It’s been used against me…I’m deemed not mentally stable. I have great concerns with this information being allowed to be used by commercial companies’ (P28).

All risks were considered more significant when sharing children’s health data, due to concerns that, on reaching adulthood, children might disagree with decisions made on their behalf. Many were worried about the future, unknown risks due to advances in AI. Some suggested offering ‘child[ren] an opportunity to opt out in the future, should they want to’ (P21), although this solution was acknowledged to be resource-intensive and potentially impractical.

Participants were apprehensive about the rapid pace of AI development and potential ‘unintended consequences’ (P11) of health data sharing for AI research. They were concerned that the speed of research outpaces the understanding of associated risks: ‘it [AI] would go a lot faster and potentially a lot further before something critical was understood’ (P13). Many believed that ‘AI has an image problem’ (P39) and that, as an emerging technology, public understanding and acceptance would be limited. There was a consensus that, for individuals to make an accurate and informed risk-benefit calculation in sharing health data, clear explanation of the added benefit of AI to the research project was required.

##### Anticipated benefits and motivations for participation

There was widespread belief that integrating AI into healthcare systems would improve systems efficiency, diagnosis and management: ‘how can you argue against that, saving lives?’ (P41). Participants were motivated by altruism; some directly linked sharing their health data to the ‘greater good’ (P38): ‘Maybe in the future, we’ll have a cure for cancer…because of people like us contributing’ (P18).

Some, having benefited from recent research, felt duty-bound to contribute: ‘I wouldn’t be here now but for people consenting’ (P12). Others were compelled by inadequate research on their condition, or past negative experiences in seeking diagnosis: ‘I just wish that there was something like that out there for me, when I was fighting to get a diagnosis’ (P20).

#### Informed consent as a foundation for trust

Consent was viewed as a key mechanism for enabling informed decision-making, acting as the bridge between general perceptions of data risk and personal risk-benefit calculations. Two subthemes were developed: (A) content of consent, emphasising the importance of clarity, comprehensiveness and individualised choice and (B) process of consent, highlighting timing and appropriateness of seeking consent and the person who obtains consent.

##### Content of consent

Participants emphasised the importance of clear, comprehensive information regarding the purpose of the proposed research, a study definition of AI, anonymisation methods, data management, the volume and type of data shared and who would access the data. Some sought additional details about the ethical approval process, data storage and security, although this was tempered by an acknowledgement that others may not find this necessary: ‘although it should be comprehensive, I don’t think it should be so many words [on information sheets] so you don’t actually read it, it’s got to be clear, concise’ (P36). Personalised consent was proposed, giving individuals the option to choose the level of detail included. In the context of research databases, participants wanted control over data retention periods, the type of data shared with different research bodies and conditions for sharing data: ‘It could be you don’t have to opt-in for everything…some won’t want to share information about certain conditions, or with commercial companies but may be happy for other things’ (P19).

##### Process of consent

Participants highlighted the importance of who obtains consent and when it is sought. Clinicians were generally viewed as more trustworthy than researchers or commercial representatives, who were seen as ‘trying to grab whoever they can…making money from every signature’ (P36). However, many questioned whether clinicians had sufficient expertise to explain AI-related risks and benefits: ‘you’re trusting the doctor to have fully understood the implications here, and I’m not sure that will be the case’ (P31). Others raised concerns about accountability, arguing that ‘doctors shouldn’t be responsible…it should be somebody who’s going to put their head on the block’ (P32).

Participants opposed obtaining consent during emotionally difficult moments, such as distressing consultations*: ‘*you’ve just been through a traumatic experience. I’m not sure I’d react very well’ (P35). Many were concerned about being pressured into consenting: ‘I don’t think the intentions are necessarily exploitative, but there’s emotional pressure on [participants]’ (P7).

Participants proposed a ‘cooling-off’ (P40) period with a follow-up discussion: ‘people need to take that information home and come back with questions, talk things through and get a better picture’ (P32). Other proposals included allowing participants ‘to leave the programme, have your information withdrawn’ at any time (P37), or implementing a government-facilitated opt-in/opt-out system.

## Discussion

### Main findings

This study presents an analysis of eight UK-based focus groups, exploring public perceptions of health data sharing for AI research. The three central themes in this study provide a framework to understand public trust, acceptance and willingness to share personal health data for AI research.

Perceived risk was a major determinant of participation (Theme 1). Although anonymisation was considered essential, participants doubted its reliability, particularly in the context of rare conditions or data linkage. They preferred targeted, purpose-specific sharing over large-scale data aggregation. Trust varied by data recipient. Universities were generally viewed as aligned with public interest, whereas commercial organisations were met with scepticism, seen as profit-driven and untrustworthy. However, this mistrust was not absolute: participants described a pragmatic, highly conditional acceptance of commercial involvement when patient benefit was clearly demonstrated. This highlights the need for transparent and stringent data management practices to build and maintain public trust, in line with expectations from prior studies.^38^

Decisions to share health data were shaped by personal context and perceived benefits (Theme 2). Altruism, civic duty and past experiences of healthcare inequity motivated participation, aligning with previous research.^39 40^ However, privacy concerns, fear of discrimination and potential misuse of sensitive health information, particularly for children, tempered this willingness. Apprehension about the rapid development of AI technologies further complicated this risk-benefit analysis. Previous studies investigating public perceptions of AI have found limited understanding and variable, often negative attitudes.^13 41^

Consent was central to public participation, enabling individuals to assess the specific risks of a study and relate them to their personal circumstances (Theme 3). To support meaningful decision-making, consent must be clear and comprehensive. Tailored approaches, such as allowing participants to opt-in/out of specific data uses, or request varying levels of detail, were viewed favourably by participants seeking greater control over personal information. Equally important was the consent process. Participants emphasised avoiding emotionally vulnerable moments and advocated for a ‘cooling-off’ period and ongoing opportunities for withdrawal.

Our findings align with and extend existing evidence. Studies from the UK, Canada and the USA have consistently shown that members of the public are more comfortable sharing data with public-sector or academic institutions and substantially less willing to share with commercial companies.^15 19 33^ Anonymisation was typically viewed as a necessary condition for health data sharing; however, willingness declines with any perceived risk of re-identification.^15 33 42^ Concerns about data sensitivity, security breaches and data misuse are similarly documented.^15^ Our findings provide further, rich contextual insight into how these factors interact, and how pragmatic acceptance can coexist with broader mistrust, particularly in relation to commercial entities.

### Strengths and limitations

This study has several strengths. The design, delivery, and analysis were co-produced with PPI contributors, on the basis of previous co-produced research on the same topic. This ensured that the research question, topic guide, and interpretation were grounded in public priorities and concerns. We recruited a broad range of participants across the UK, with representation from different age groups, genders, regions, and ethnic and health backgrounds, to maximise diversity of opinion. The focus group format encouraged participants to engage in a dialogue with one another, often prompting lively debate and facilitating deeper exploration of shared and contrasting viewpoints. The use of plausible scenarios helped anchor discussions and provided rich insight into how people reason about risk, trust and consent in practical contexts, rather than purely abstract or hypothetical terms.

This study also has several limitations. While our study represented a wide range of people across the UK, we excluded participants who were not fluent in English, limiting our reach to some ethnic minority groups. The sample was skewed towards more affluent and educated individuals, with a median household income of £50–69 000/year. By conducting focus groups online, we excluded people without Internet access. We did not sample participants by previous research participation, which may have influenced familiarity and comfort with involvement in health data research and consent processes. An important characteristic of our sample was the high proportion (73%) of participants living with chronic health conditions. This may limit transferability, as their experiences could heighten awareness of re-identification or discrimination risks. However, this group often engages more with healthcare systems and may be more vulnerable to discriminatory data misuse, making their perspectives valuable for identifying concerns relevant to populations likely to share more data and potentially benefit from, or be harmed most by data sharing.

Finally, we only included participants from the UK, and it is likely that there are differences in opinion internationally, depending on the structure of healthcare systems and cultural context.^19^ Limited participation from certain demographic groups poses risks to equitable representation, and therefore performance and fairness of AI systems. Future comparative studies across multiple countries, and targeting under-represented sociodemographic groups, could identify universal and culturally specific concerns, informing policy and practice in AI healthcare research.

### Study implications

#### Data governance and policy

This study highlights the importance of robust, transparent governance as the foundation of public trust in health-data sharing for AI. Participants emphasised safeguards, such as effective anonymisation, or clear explanation of its limits, controlled data access and consequences for misuse. These expectations align with current policy developments, including NHS England’s Secure Data Environments, which standardise health data access under NHS oversight, and the European Health Data Space (EHDS) Regulation, which aims to establish cross-border access to EHR for primary and secondary use of data, through a single infrastructure.^43 44^

Our findings support these policy directions. Trust was conditional on demonstrable patient benefit, appropriate custodianship and strict access controls. Governance models perceived as opaque, overly permissive or commercially driven without patient benefit were viewed negatively. Policy that assumes blanket public support for large-scale data reuse risks overestimating the social licence required for AI development.

#### Research design, communication and consent

Expectations for transparency and control have direct implications for the design and delivery of AI-related research. Participants preferred clear, study-specific definitions of AI, the proposed use case and added value. They were pragmatic about security risks and favoured candid acknowledgement of uncertainties over reassurance alone.

We therefore recommend that researchers offer tailored consent processes, enabling participants to choose the level of detail they receive. Data retention and destruction policies should be clearly communicated, including where derived datasets may persist. Cooling-off periods, or opportunities for later withdrawal, should be integrated into consent processes.

### Future research

Future research could build on these findings in several ways. First, cross-national comparative studies may identify similarities and differences in attitudes towards health data sharing across healthcare systems and cultural contexts. Comparing perspectives in the UK, with those in EU countries operating under the EHDS, and in settings without universal healthcare, could help distinguish universal concerns from context-specific priorities and inform local governance frameworks or facilitate international AI research.

Second, targeted engagement with under-represented or digitally excluded groups, including minority ethnic communities and people with limited English proficiency, is essential to avoid reinforcing existing exclusions. Related to this, empirical testing of tailored consent models, such as tiered consent, is recommended to evaluate their feasibility, clarity and acceptability for diverse populations.

Finally, further research should examine public involvement in ongoing research and governance, including patient advisory groups. Longitudinal studies could also examine how public perceptions evolve as AI becomes more visible in routine clinical care.^19^

## Conclusion

The integration of AI could bring huge advantages to healthcare systems. AI development and implementation necessitate large-scale data sharing, but public trust in such initiatives is contingent on the factors identified in this study. Researchers and policymakers must prioritise robust anonymisation techniques, transparent data management practices and clear communication strategies that respect individual preferences and provide sufficient information for informed consent. By addressing these factors, it is possible to enhance public acceptability and participation in health data sharing for AI research, contributing to the development of technologies that are effective and representative of population diversity.

## Data Availability

No data are available. All data relevant to the study are included in the article or uploaded as supplementary information. Focus group transcripts contain information about individual participants with a risk of re-identification; we are not able to share raw transcripts.
