# Supplementary material for: Public perceptions of health data sharing for artificial intelligence research: a qualitative focus group study in the UK
Source: BMJ Digit Health Ai. 2026 Feb 9;2(1):e000239. doi: 10.1136/bmjdhai-2025-000239 (PMC13528373; doi:10.1136/bmjdhai-2025-000239)
Supplement: Supplementary file 1 [file bmjdhai-2-1-bmjdhai-2025-000239-s001.pdf]

Appendix

Reflexivity statements 1
Topic guide 4
Introductions 4
Questions 5
Scenarios 6
Scenario 1 6
Scenario 2 7
Scenario 3 8
Close 8
SRQR checklist[1] 8
Participant demographics 11
Supplementary references 12

Reflexivity statements

RK is a South-East Asian female undertaking a DPhil in musculoskeletal sciences at the University of Oxford, having taken time out from training in Plastic and Reconstructive surgery. Prior to this study, RK undertook formal training in qualitative research methods, including focus group facilitation. She has a professional and personal interest in the application of artificial intelligence in healthcare, and sought feedback from all other members of the research team on her interpretations of the focus group data, particularly those with a sceptical attitude towards AI.

CO is a male researcher of mixed ethnicity (South-East Asian/White Irish), working as an academic doctor in Oxford. He has training in ethnographic qualitative methods and in focus group facilitation. CO's parents work as management consultants in the public sector, which contributed to his longstanding interest in how new technologies can improve services and outcomes. This background underpins his professional interest in the use of artificial intelligence (AI) to increase efficiency and improve outcomes in healthcare.

Although CO has a generally pro-innovation outlook, he is aware of and sympathetic to public concerns about AI, particularly around the use of personal data. He is interested in how regulation and public communication can support the responsible use of AI. Recognising his own positive attitude towards AI, CO took care to facilitate balanced discussions in the focus groups. He avoided leading questions, followed a pre-prepared topic guide, and ensured that participants with differing views had equal opportunities to contribute. During the analysis, CO regularly reviewed his coding for evidence of bias and patterns that might reflect his own assumptions. He discussed his emerging themes with other members of the research team, including colleagues who take a more cautious view of AI and data sharing, and incorporated their feedback into the final interpretation.

JS is a white British semi-retired female. She has a professional background in both health and education and has been involved in Patient and Public Involvement for the last 4 years. She is also a carer for 2 family members with rare long term health conditions which have a major impact on their lives. There is little research into these conditions and even less specific treatment. This has fuelled an interest into how AI can accelerate medical understanding of rare conditions. JS is relatively new to research and AI so can identify with many of the hesitations expressed by those in the focus groups.

RH is a white British female with a background in political science and healthcare policy. She currently works in the health security sector and has a particular interest in health data practices and governance. Her involvement in PPI began three years ago, motivated by her own experiences within UK healthcare. She holds a cautiously optimistic view of AI in healthcare, while remaining sceptical of some of the claims surrounding its potential. She aimed to bring a policy-oriented and governance lens to the analysis, ensuring that participants' perspectives were interpreted in the context of broader debates about trust, data use, and public accountability.

BP is an Associate Professor in medical image analysis and machine learning at the Big Data Institute, University of Oxford. His work centres on the development and application of AI

methods for medical imaging. Coming from a technical background, he recognised that his assumptions about AI innovation could differ from public experiences and priorities. He aimed to offer insight into current and emerging technologies while being conscious not to over-privilege a technological viewpoint, and to ensure that participants' perspectives guided interpretation.

GSC is a Professor of Medical Statistics at the University of Birmingham, with particular interest in the methodology, and transparent reporting, of the development and evaluation of prediction models. He has led the development of multiple reporting guidelines for AI-based studies (TRIPOD-AI, CONSORT-AI, SPIRIT-AI, STARD-AI, DECIDE-AI, QUADAS-AI, and PROBAST-AI). He holds a sceptical position on the claims of healthcare AI, and aimed to provide a rigorous methodological perspective throughout this study, challenging assumptions, clarifying analytical choices, and ensuring that interpretations were grounded in evidence rather than enthusiasm.

DF is Professor of Plastic and Reconstructive Surgery at Oxford University, with a special interest in hand surgery and microsurgery. His research work encompasses the use of big data, and he has a strong belief in using data ethically in order to maintain public trust and therefore facilitate more academic research, and progress within healthcare. He has some scepticism as to the current usefulness of AI in healthcare, but recognises the future potential. He has supported and encouraged RK in pursuing this research project in order to help foster responsible, trustworthy introduction of AI into healthcare settings.

ET is an experienced qualitative researcher in health sciences. She leads a theme of work on patient experience of illness and injury and staff experience of care provision. Insights gained from active research participation using a range of methodology and supervision of students underpinned her contribution. In this study, understanding of how participants made sense of data sharing and weighed up the risks was of particular interest.

Topic guide

Introductions

Hello everyone, thank you very much for joining us today. My name is Rachel, and I am a PhD student and researcher based at the University of Oxford. I've got a background as a surgeon, and I'm interested in how artificial intelligence, or AI, is used or should be used in healthcare. I'll let Judi/Rosie/Ciaran introduce themselves.

The reason that we are all here today is to explore and try to understand what people in the UK think about artificial intelligence, and in particular using health data for AI research. We think it's really important to include public voices in this debate, and we're looking forward to hearing what you think.

Just a few housekeeping things.

It's important to stress that there aren't any right or wrong answers, but just different points of view. There may be times where you disagree with others in the group, or others disagree with you. Please don't be shy to voice your opinion but we should keep all discussions respectful and make sure that everyone gets a chance to speak. Do speak freely, or if you feel that it would be helpful, please put your hand up on the screen. So that we are all on the same page, please only talk one at a time and avoid having 'side -discussions'.

Judi/Rosie/Ciaran and I are here to moderate the group and make sure that we get to listen to everyone's point of view. If you have any questions or need help with technical issues please let us know in the chat bar on the side.

We are video-taping the focus group today, and that is to help us to transcribe the focus group into a written 'script' afterwards. We will destroy the video recording straight after. If you can, please keep your camera on and mute yourself when you aren't speaking! If you need to get up for food or to go to the bathroom just let us know when you leave and when you return.

Everything that you say today will be kept only for research, and your participation is confidential- although we're on a first name basis today, we won't share any names in our reports. Please don't share anything personal or identifiable about others in the group afterwards, for example if someone shares something from their own health experiences.

If at any point you feel uncomfortable, you're welcome to leave completely, or re-join at any time.

OK, so just to get started, let's find out some more about each other. We'll go around the 'room' and please tell us your name and where you're from/share why you're here if you like!

Questions

First of all, we'd like to know what comes to mind when you think about 'artificial intelligence', and what do you think has contributed to that.

Ok, great! Before we continue, we'll just explain what we mean when we talk about 'AI' and 'health data'- this might be different to what you have told us already, but this is just so that we have some common ground.

By 'AI' we mean computer systems that can learn and solve problems, almost like a person. They learn from large amounts of information, or data, and learn to find patterns in that data. For example, in health, this might be learning how to diagnose skin cancer.

By 'health data', we mean any information that is related to people's physical or mental health. This could be anything from written records like letters or electronic notes, images such as x-rays, blood test results or observations like your heart rate or blood pressure.

Does anyone have any questions at this point, before we move on to the main discussion?

Scenarios

Scenario 1

I am just going to start by giving you a short vignette.

Anna has gone to see a dermatologist (skin doctor) in hospital. She has had a suspicious looking mole removed from her arm and sent for testing, and has arrived to find out the results. Her doctor tells her that the mole was benign (not cancer) and no further treatment is needed.

Before she leaves, her doctor asks her if her health data can be shared to develop and test an AI to help diagnose skin cancer. The data that they would like includes some information about her (for example, her age, 67, and ethnic background, White British), the photograph of the mole before it was removed, and the results of the laboratory test that shows that it is not cancer.

The project is being run by a group of researchers at the local University, alongside some of the doctors in the hospital. Her data will be transferred from the hospital to the

computers in the University.

Her doctor reassures her that her health data will be anonymised so that she could not be identified from her data. Her data will only be used for this research project and only researchers who are working on the project will have access to her data. Her doctor then tells Anna that her data will be destroyed at the end of the project.

The doctor asks Anna to sign a consent form.

Scenario 2

Thomas has made an appointment with his GP for help with depression and anxiety.

At the end of his appointment, his GP asks if he would be willing to share his health data for an AI research database. This would be all of Thomas' records at the GP, including his age, profession, ethnicity, and medical history.

His GP explains that the practices across the local area are being asked to share their records to contribute to a large, anonymous health database. This database is under the control of a research group based at a university, but the data will be stored on NHS computers.

Anyone wanting to use the database for AI research would have to apply to the group beforehand to gain access. This would include researchers and could include commercial companies. The research database is reviewed every five years to check if it is still helping research and that the data is held securely. However, there isn't a maximum time that the data will be kept for.

Some of the projects that the database has contributed to includes: an AI programme to identify people at risk of developing kidney disease, an AI programme to help to schedule appointments and an AI programme that helps GPs to manage diabetes.

NB. Change variable – no consent sought, only opt-out

Scenario 3

Amina and Mark are in hospital with their 7-year-old child, Ellie. Ellie is just about to be discharged after a short stay with asthma. As they are about to leave, a research nurse approaches them and asks if they would be willing to share Ellie's health data for an AI research project.

A large company based in another country has made an AI programme that can predict which children will have to be hospitalised for asthma, using information from GP records and test results and observations done in A&E. Ellie's data would be used to train and test the AI for patients in the UK. The health data that the company and researchers would need includes all of Ellie's GP, health visitor records, and hospital records, including her medication list and vaccination status.

Ellie's health data would be kept in the UK but representatives from the company would have access to her data. Afterwards, Ellie's data would be deleted.

NB – change variable; data kept indefinitely

Close

Thank you very much for your time today, it has been great to meet you all. If you've consented to updates on the project we will let you know of our results, otherwise all the best and thank you for such a good discussion!

SRQR checklist[1]

	Page/line no(s).
**Title and abstract**	

**Title** - Concise description of the nature and topic of the study Identifying the study as qualitative or indicating the approach (e.g., ethnography, grounded theory) or data collection methods (e.g., interview, focus group) is recommended	Title; Page 1
**Abstract** - Summary of key elements of the study using the abstract format of the intended publication; typically includes background, purpose, methods, results, and conclusions	Abstract; Page 1

Introduction

**Problem formulation** - Description and significance of the problem/phenomenon studied; review of relevant theory and empirical work; problem statement	Introduction, paragraphs 1 and 2
**Purpose or research questio**n - Purpose of the study and specific objectives or questions	Introduction; paragraph 3

Methods

**Qualitative approach and research paradigm** - Qualitative approach (e.g., ethnography, grounded theory, case study, phenomenology, narrative research) and guiding theory if appropriate; identifying the research paradigm (e.g., postpositivist, constructivist/ interpretivist) is also recommended; rationale**	Methods; data analysis
**Researcher characteristics and reflexivity** - Researchers' characteristics that may influence the research, including personal attributes, qualifications/experience, relationship with participants, assumptions, and/or presuppositions; potential or actual interaction between researchers' characteristics and the research questions, approach, methods, results, and/or transferability	Methods; researcher characteristics; Appendix
**Context** - Setting/site and salient contextual factors; rationale**	Methods; Recruitment and Sampling; Focus groups

**Sampling strategy** - How and why research participants, documents, or events were selected; criteria for deciding when no further sampling was necessary (e.g., sampling saturation); rationale**	Methods; Recruitment and Sampling
**Ethical issues pertaining to human subjects** - Documentation of approval by an appropriate ethics review board and participant consent, or explanation for lack thereof; other confidentiality and data security issues	Methods; Recruitment and Sampling; Ethical approval
**Data collection methods** - Types of data collected; details of data collection procedures including (as appropriate) start and stop dates of data collection and analysis, iterative process, triangulation of sources/methods, and modification of procedures in response to evolving study findings; rationale**	Methods; Focus Groups
**Data collection instruments and technologies** - Description of instruments (e.g., interview guides, questionnaires) and devices (e.g., audio recorders) used for data collection; if/how the instrument(s) changed over the course of the study	Methods; Topic guide; Appendix
**Units of study** - Number and relevant characteristics of participants, documents, or events included in the study; level of participation (could be reported in results)	Results; Participants
**Data processing** - Methods for processing data prior to and during analysis, including transcription, data entry, data management and security, verification of data integrity, data coding, and anonymization/de-identification of excerpts	Methods; Data analysis
**Data analysis** - Process by which inferences, themes, etc., were identified and developed, including the researchers involved in data analysis; usually references a specific paradigm or approach; rationale**	Methods; Data analysis
**Techniques to enhance trustworthiness** - Techniques to enhance trustworthiness and credibility of data analysis (e.g., member checking, audit trail, triangulation); rationale**	Methods; data analysis

Results/findings

**Synthesis and interpretation** - Main findings (e.g., interpretations, inferences, and themes); might include development of a theory or model, or integration with prior research or theory	Results (all); Table 1, Figure 1
**Links to empirical data** - Evidence (e.g., quotes, field notes, text excerpts, photographs) to substantiate analytic findings	Results; Themes

Discussion

**Integration with prior work, implications, transferability, and contribution(s) to the field -** Short summary of main findings; explanation of how findings and conclusions connect to, support, elaborate on, or challenge conclusions of earlier scholarship; discussion of scope of application/generalizability; identification of unique contribution(s) to scholarship in a discipline or field	Research in Context panel; Discussion; paragraphs 1-4
**Limitations** - Trustworthiness and limitations of findings	Discussion; paragraph 5

Other

**Conflicts of interest** - Potential sources of influence or perceived influence on study conduct and conclusions; how these were managed	Conflicts of Interest
**Funding** - Sources of funding and other support; role of funders in data collection, interpretation, and reporting	Funding

Participant demographics

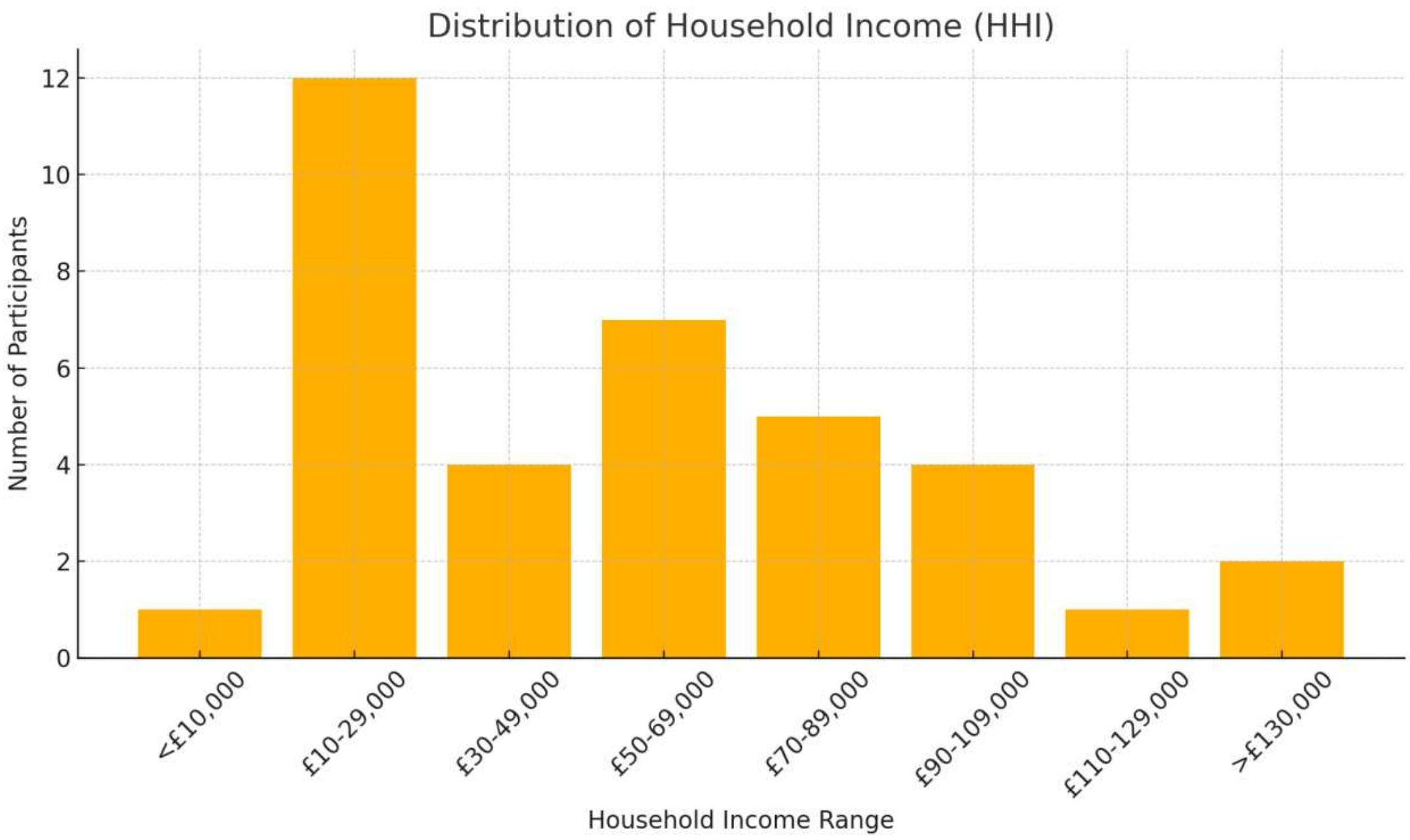

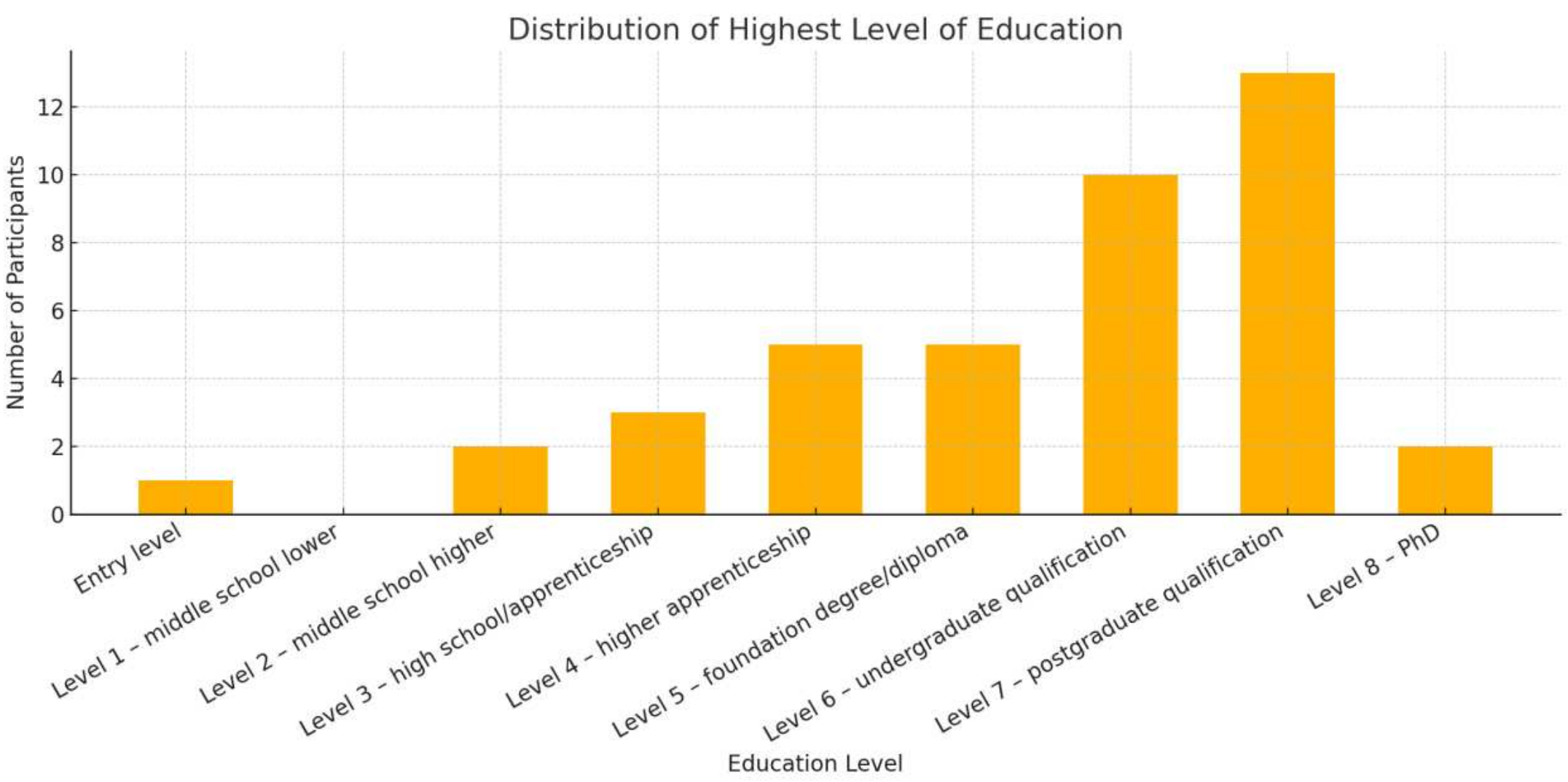

Supplementary references

1. O'Brien BC, Harris IB, Beckman TJ, Reed DA, Cook DA. **Standards for reporting qualitative research: a synthesis of recommendations.** *Academic Medicine*, Vol. 89, No. 9 / Sept 2014 DOI: 10.1097/ACM.0000000000000388
