# Supplementary material for: Public perceptions of health data sharing for artificial intelligence research: a qualitative focus group study in the UK
Source: BMJ Digit Health Ai. 2026 Feb 9;2(1):e000239. doi: 10.1136/bmjdhai-2025-000239 (PMC13528373; doi:10.1136/bmjdhai-2025-000239)
Supplement: Supplementary file 2 [file bmjdhai-2-1-bmjdhai-2025-000239-s002.pdf]

Supplementary Table 1. Focus group demographics

		FG1	FG2	FG3	FG4	FG5	FG6	FG7	FG8	Total
**Number of participants**		5	4	5	6	4	7	5	5	41
**Female participants (%)**		3	1	3	3	3	3	2	4	22 (53.7%)
**Mean age in years (range)**		47.2 (38-60)	46.8 (25-72)	65.4 (59-75)	48.0 (23-78)	70.8 (64-77)	60.4 (39-77)	63.0 (57-69)	53.4 (25-67)	56.7 (25-78)
**Ethnicity**	**White ethnicity (%)**	4	2	3	5	4	5	5	4	32 (78.0%)
	**Asian ethnicity (%)**	1		1	1				1	4 (9.8%)
	**Other ethnicity (%)**		2	1			2			5 (12.2%)
**Presence of existing health condition (%)**	**No**	2	2	1	1		1	2	2	11 (26.8%)
	**Chronic health condition (3 or more months' duration)**	3	2	4	5	4	6	3	3	30 (73.2%)

**Household income per annum (%)**	**<£10,000**							1		1 (2.4%)
	**£10-29,000**	2		3	2	1	3	1		12 (29.3%)
	**£30-49,000**	1		1	1	1				4 (9.8%)
	**£50-69,000**	1	1		1	2	2			7 (17.1%)
	**£70-89,000**		1	1			1	2		5 (12.2%)
	**£90-109,000**		1		1		1	1		4 (9.8%)
	**£110-129,000**				1					1 (2.4%)
	**>£130,000**	1	1							2 (4.9%)
**Highest level of education (%)**	**Entry level**	1								1 (2.4%)
	**Level 1 – e.g., middle school lower grades**									0 (0.0%)

	**Level 2 – e.g., middle school higher grades**							1	1	2 (4.9%)
	**Level 3 – e.g., high school or apprenticeship**	1					2			3 (7.3%)
	**Level 4 – e.g., higher apprenticeship**				3	1	1			5 (12.2%)
	**Level 5 – e.g., foundation degree or diploma**	1	1	2				1		5 (12.2%)
	**Level 6 – e.g., undergraduate**	2	1	3		1	2		1	10 (24.3%)

	**qualification**									
	**Level 7 – e.g,. postgraduate qualification**		2		3	2	1	2	3	13 (31.7%)
	**Level 8 – e.g., PhD**						1	1		2 (4.9%)
